# Short RNA half-lives in the slow-growing marine cyanobacterium *Prochlorococcus*

**DOI:** 10.1186/gb-2010-11-5-r54

**Published:** 2010-05-19

**Authors:** Claudia Steglich, Debbie Lindell, Matthias Futschik, Trent Rector, Robert Steen, Sallie W Chisholm

**Affiliations:** 1 Massachusetts Institute of Technology, Department of Civil and Environmental Engineering, Cambridge, MA 02139, USA; 2 University of Freiburg, Faculty of Biology, D-79104 Freiburg, Germany; 3 Technion - Israel Institute of Technology, Faculty of Biology, Haifa 32000, Israel; 4 University of Algarve, Institute for Biotechnology and Bioengineering, Centre for Molecular and Structural Biomedicine, 8005-139 Faro, Portugal; 5 Humboldt University, Institute for Theoretical Biology, Charité, 10115 Berlin, Germany; 6 Harvard Medical School, Department of Genetics, Biopolymers Facility, Boston, MA 02115, USA; 7 PerkinElmer Life and Analytical Sciences, Waltham, MA 02451, USA

## Abstract

**Background:**

RNA turnover plays an important role in the gene regulation of microorganisms and influences their speed of acclimation to environmental changes. We investigated whole-genome RNA stability of *Prochlorococcus*, a relatively slow-growing marine cyanobacterium doubling approximately once a day, which is extremely abundant in the oceans.

**Results:**

Using a combination of microarrays, quantitative RT-PCR and a new fitting method for determining RNA decay rates, we found a median half-life of 2.4 minutes and a median decay rate of 2.6 minutes for expressed genes - twofold faster than that reported for any organism. The shortest transcript half-life (33 seconds) was for a gene of unknown function, while some of the longest (approximately 18 minutes) were for genes with high transcript levels. Genes organized in operons displayed intriguing mRNA decay patterns, such as increased stability, and delayed onset of decay with greater distance from the transcriptional start site. The same phenomenon was observed on a single probe resolution for genes greater than 2 kb.

**Conclusions:**

We hypothesize that the fast turnover relative to the slow generation time in *Prochlorococcus *may enable a swift response to environmental changes through rapid recycling of nucleotides, which could be advantageous in nutrient poor oceans. Our growing understanding of RNA half-lives will help us interpret the growing bank of metatranscriptomic studies of wild populations of *Prochlorococcus*. The surprisingly complex decay patterns of large transcripts reported here, and the method developed to describe them, will open new avenues for the investigation and understanding of RNA decay for all organisms.

## Background

The rate of degradation of RNA is an important factor in the regulation of gene expression. It is well known that stress conditions, such as the presence of antibiotics, nutritional stress, and transitions in growth phase, cause a dramatic change in the rate of mRNA turnover for a subset of genes within a particular organism [1-3]. The stability of RNA encoded by certain genes can also be greatly affected by the growth rate of the cell [3,4]. However, a genome-wide analysis showed that the half-lives of the vast majority of *Escherichia coli *transcripts do not differ with growth rate [5], suggesting an inherent median global half-life for a certain organism.

Whole genome half-life analyses comparing very different organisms, such as fast-growing bacteria and slower-growing eukaryotes, however, initially suggested that global RNA decay rates correlate with the intrinsic growth rate of the organism: ranging from minutes to hours in bacteria [5-7] and hours to days for eukaryotes [8-10]. The investigation of global RNA half-lives of archaea, which have intermediate growth rates, led to conflicting conclusions, with one study showing global half-lives similar to bacteria [11] and another showing considerably longer half-lives [12]. To help resolve this issue we examined the global RNA half-live in the slow growing marine cyanobacterium *Prochlorococcus *MED4.

*Prochlorococcus *is an abundant component of the phytoplankton in the vast oligotrophic tropical and subtropical open oceans where it contributes a significant fraction of photosynthesis [13,14]. Despite the high abundance of *Prochlorococcus *in these waters, it grows very slowly with growth rates of usually one division per day [15] and, at most, two divisions per day [16]. Complete genome sequences of 12 cultured isolates of *Prochlorococcus *are now available [17-21] and reveal that genome reduction has left a minimal inventory of protein coding regulatory genes, but the regulatory capacity of *Prochlorococcus *has been complemented with numerous small non-coding RNAs (ncRNAs) [22,23].

Changes in global gene expression profiles in the model *Prochlorococcus *strain MED4 have been studied under different light conditions [24], nitrogen and phosphorus depletion [25,26] and during bacteriophage infection [27]. In addition, metatranscriptomic data are currently being collected to characterize the physiological status of natural oceanic communities of which *Prochlorococcus *is often the dominant photosynthetic organism [28-31]. However, little is known about RNA stability in *Prochlorococcus*. This is of central importance if we are to understand the role RNA turnover plays in controlling gene expression.

## Results and discussion

### Determination of RNA half-lives and decay rates

We examined the half-lives of known and predicted mRNAs and non-coding RNAs in *Prochlorococcus *MED4 at single-gene resolution using high density Affymetrix microarrays [24]. Rifampicin, which prevents initiation of new transcripts by binding to the β subunit of RNA polymerase [32], was added to triplicate cultures. Samples were harvested at 0 minutes (before rifampicin addition), and 2.5, 5, 10, 20, 40 and 60 minutes after rifampicin addition. As shown previously in a similar microarray experiment for *E. coli *[7], the decay of RNA does not always follow an exponential curve, which deems it necessary to adjust and improve existing methods for the calculation and description of RNA decay. Thus, we applied two different approaches: the so-called 'twofold' decay step method as proposed previously by Selinger *et al. *[7] in order to determine the RNA half-life; and a new method developed here based on fitting the decay profile to two distinct phases to derive the decay rate (see Materials and methods). The latter method was more accurate to describe decay patterns of genes that displayed two distinct decay phases: either a fast decay followed by a slow decay; or an apparent initial period of constant expression or even increase in expression prior to the decay. Notably, large differences between the two methods were observed only for genes with a delayed onset of degradation or for genes with very stable half-lives (Additional file 1). For the determination of global half-lives and decay rates we excluded genes with low expression signals below a set threshold, resulting in data for 1,102 genes (including protein-, ribosomal-, tRNA, ncRNA and antisense RNA (asRNA) coding genes).

### Genome-wide RNA decay

**Figure 1:**
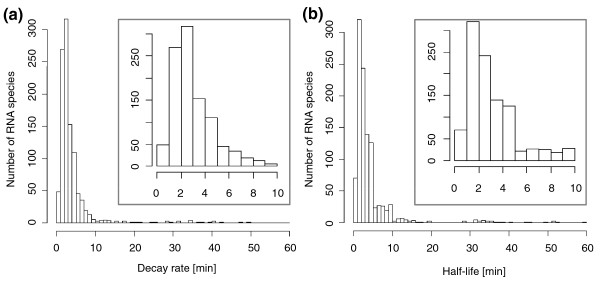
**Distribution of RNA decay rates and RNA half lives using the two phase decay step or the twofold decay step method**. **(a) **RNA decay rates. **(b) **RNA half-lives. Time rates were binned in 1-minute increments. RNAs with stabilities of more than 60 minutes are not shown. The insets show the results for transcripts with decay rates of ≤10 minutes.

The median half-life and the median decay rate of expressed genes were estimated to be 2.4 and 2.6 minutes, respectively (Figure 1). Half-lives for 80% of the genome ranged from 1.1 to 8.9 minutes. The hypothetical gene PMM1003 displayed the shortest half-life and decay rate at 33 seconds. Only 3% of all genes showed a half-life of more then 60 minutes and hence were considered to be stable (Additional file 1). The longest half-lives of protein-coding transcripts were found for *psbA *(PsbA protein D1), *amt1 *(permease for ammonium transport), *pcb *(light harvesting complex protein) and *som *(PMM1121, porin; Additional file 1). Verification of half-life calculations from microarray data with those from quantitative RT-PCR (qRT-PCR; 17 genes) showed a very high level of correlation for genes with average-to-low transcript abundance (Table 1; Additional file 2). However, half-life estimates calculated for highly expressed genes were longer when using microarray data than when using qRT-PCR measurements, indicating that half-life calculations for these highly expressed protein coding genes (only ten in the genome) were affected by microarray saturation and should be treated with caution. For example, the half-life and decay rate of *psbA *were calculated to be 40 and 70 minutes, respectively, from the microarray data but determined to be 18.5 and 16.2 minutes by qRT-PCR (Table 1). These qRT-PCR results correlate very well with what has been published previously by Kulkarni *et al. *[33], who determined a half-life of 18 minutes for *psbAI *in *Synechococcus *PCC 7942 under standard light growth conditions.

**Table 1:** Comparison of decay rates and half-lives of 17 selected genes determined from microarray data and qRT-PCR

	Microarray				qRT-PCR	
		
Gene	Cluster	Expression at time 0 [log2]	Half-life [min]	Decay rate [min]	Half-life [min]	Decay rate [min]
PMM1077	7	5.6	1.7	2.4	1.6	1.6
*dnaN*	3	6.4	1.5	1.7	3.0	2.1
*psaK*	9	12.3	4.9	5.0	5.3	4.8
*atpA*	11	10.9	12.2	4.9	6.2	3.3
*psbA*	11	14.3	40.1	71.0	18.5	16.2
*recN*	ND	4.0	3.9	5.3	2.2	6.4
*recA*	5	8.5	2.3	2.7	2.6	7.5
*ftsZ*	5	9.4	1.8	2.1	3.4	2.1
*amt1*	11	13.7	52.1	77.2	17.3	11.3
*psbD*	9	13.2	9.0	8.8	7.0	5.6
PMM1121 (*som*)	11	13.9	28.6	39.1	13.0	10.4
*pcb*	11	13.9	15.6	25.8	6.6	6.3
PMM1447	ND	3.7	59.5	18.0	40.6	4.1
*atpE*	11	13.0	13.5	24.8	15.6	4.7
*atpB*	9	10.5	4.6	3.4	8.0	4.7
*atp1*	10	10.7	5.0	2.3	2.4	2.6
16S rRNA	2	14.9	370.1	20.7	-261.3	54.4

ND, not determined.

**Figure 2:**
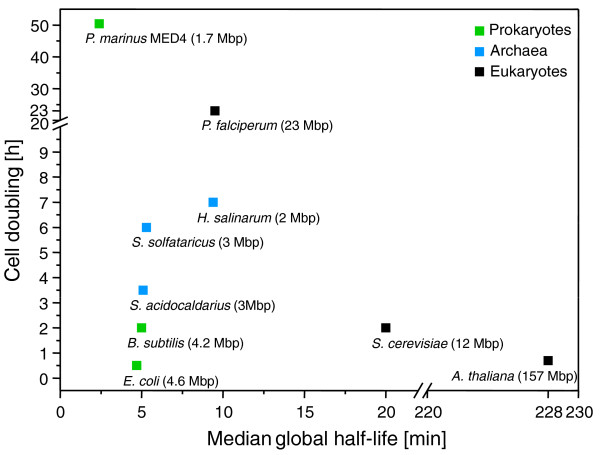
**Comparison of global half-lives and cell doubling time of selected organisms**. For all organisms the global median half-life is presented except for *Plasmodium falciperum*, for which only mean half-lives were available. Values were obtained from the following sources: *Halobacterium salinarum *[12], *Sulfolobus solfactaricus *and *Sulfolobs acidocaldarius *[11], *E. coli *[34], *P. falciperum *[54,55], *Saccharomyces cerevisiae *[56,57], *Arabidopsis thaliana *[9,58], *Bacillus subtilis *[6,59], and *Prochlorococcus marinus *(this study).

We observed a median RNA half-life of 2.4 minutes for *Prochlorococcus *MED4, which is considerably shorter than for other bacteria and archaea investigated so far (Figure 2): approximately 5 minutes for *E. coli, Bacillus subtilis, Sulfolobus solfataricus *and *Sulfolobus acidocaldarius *and 10 minutes for *Halobacterium salinarum *[6,7,11,34]. This is despite a significantly longer generation time of over 24 hours for *Prochlorococcus *versus less than 2 hours for the other bacteria and 4 to 7 hours for the archaea (Figure 2). These combined results indicate that global half-lives do not correlate directly with growth rates even within the eubacteria let alone across all three kingdoms of life. Rather, half-lives in the minutes range for eubacteria and archaea suggest an intrinsic chemical response that is similar for both bacteria and archaea to ensure rapid RNA turnover. These conclusions differ from those made by Hundt *et al. *[12] to explain the longer global half-life that they found for *H. salinarum *relative to faster growing bacteria as well as to archaea with similar doubling times (with a half-life of 10 minutes for *H. salinarum *compared to approximately 5 minutes for the other prokaryotes; Figure 2). On the one hand, the authors [12] suggested that faster growth rates in bacteria explain their more rapid half-lives, and on the other hand they invoke higher growth temperatures (of 79°C) as a potential cause for reduced RNA stability for the *Solfolobus *species. However, clearly these arguments cannot be invoked here as *Prochlorococcus *cells divide only once a day [15], grow optimally at about 25°C [35], yet have a global half-life considerably shorter than those of other bacteria and archaea.

High rates of RNA turnover are likely to facilitate the rapid adaptation of *Prochlorococus *to environmental change in the oceans and may help compensate for its minimal regulatory capacity. This is even more pronounced in relation to their slow growth as the rapid metabolic response achieved relative to growth rate would be considerably greater than for fast growing organisms. Furthermore, the fast recycling of nucleotides through rapid RNA turnover may help save resources and compensate for the scarcity of nutrients like phosphorus and nitrogen in the nutrient poor oligotrophic waters in which *Prochlorococcus *is so abundant.

### Correlation of RNA stability and gene product function

**Figure 3:**
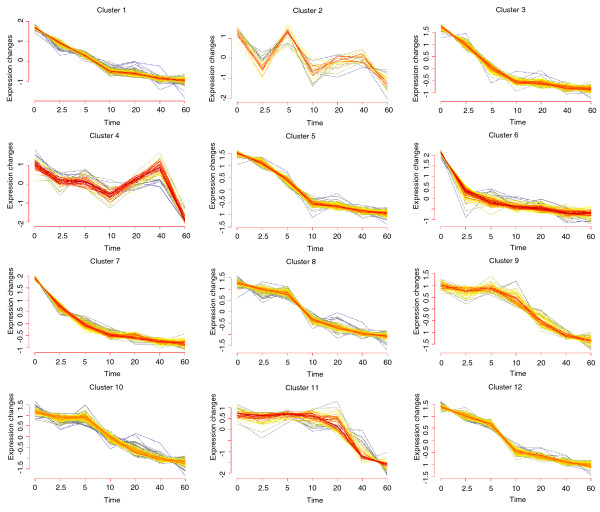
**Expression profiles of 12 clusters determined by Mfuzz**. In red are genes that are well supported within the cluster (that is, high fuzziness score) and in grey genes with weak support. Cluster 6 contains genes with the shortest half-lives and decay rates and cluster 11 highly expressed genes with long half-lives. Clusters 2 and 4 are highly enriched in genes coding for tRNAs, rRNAs and ncRNAs.

Recent studies indicate a potential correlation between RNA degradation rates and their functional role [6,34]. To address this question for *Prochlorococcus *we performed soft clustering [36] and identified 12 clusters with distinct decay profiles containing between 20 and 139 members per cluster (Figure 3). We used the functional gene categories assigned according to CyanoBase [37] to assess the significance of enrichment of functionally related genes within a cluster. In general, most clusters were not enriched for particular functions. For example, cluster 6 contains genes with the shortest half-lives and decay rates but without any accumulation in genes with the same function. However, some clusters did show enrichment for certain gene types. In particular, clusters 2 and 4 consist of genes with high RNA stability and are significantly enriched in genes coding for tRNAs and rRNAs (*P*-values ≤ 1e^-16^).

We wondered whether such long half-lives for RNA genes is related to their function in protein translation or is inherent to non-protein coding genes. We therefore investigated the half-lives of ncRNAs in *Prochlorococcus *- genes that do not code for proteins but function as regulators on the RNA level in the cell [23,38]. Table 2 shows decay rates determined for all expressed ncRNAs and asRNAs during the time course (excluding tRNAs and rRNAs). Interestingly, many of these RNAs displayed short decay rates of less than a minute to more than an hour with a median decay rate of 3.3 minutes, thus behaving like protein-coding genes. Those with longer decay rates are members of clusters 2 or 4 and represent housekeeping RNAs like *ssrA *(6S RNA), *rnpB*, *ffs *(SRP RNA) and *ssrS *(tmRNA). These findings suggest that the half-life of ncRNA is related to function rather than being inherent to non-protein coding genes. The functions of ncRNAs *Yfr1 *to *Yfr21 *[22,23] are unknown. However following from the argument above, the other long-lived ncRNAs *Yfr2*, *Yfr4*, *Yfr5 *and *Yfr16 *may also be involved in general processes in the cell. All of the stable ncRNAs are members of cluster 4 whereas the remaining ncRNAs and asRNAs are dispersed among other clusters. Thus, functional class correlates well with half-life in *Prochlorococcus *for tRNAs, rRNAs as well as for some ncRNAs.

**Table 2:** Decay rates of expressed ncRNAs and asRNAs

ncRNA/asRNA	Decay rate [min]
*rnpB *(RNase P sRNA)	>20
*ffs *(SRP RNA)	>20
*ssrA *(tmRNA)	>20
*ssrS *(6S RNA;Yfr7)	>20
Yfr4	>20
Yfr5	>20
Yfr2	>20
asRNA_04601	>20
Yfr16	>20
Yfr8	19.7
Yfr14	11.6
asRNA_17331	8.4
asRNA_17181	7.8
ncRNA_Yfr9	6.9
asRNA_15721	4.9
Yfr11	4.8
asRNA_04001	4.2
Yfr6	4.0
asRNA_38	3.5
asRNA_00641	3.4
asRNA_17971	3.2
Yfr1	3.1
asRNA_07401	2.3
Yfr19	2.2
Yfr13	2.2
asRNA_03431	2.0
Yfr20	2.0
asRNA_02731	1.7
asRNA_18171	1.6
Yfr21	1.5
asRNA_15701	0.9

**Figure 4:**
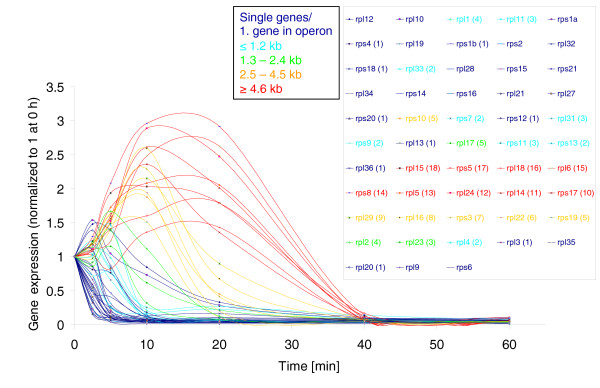
**RNA decay profiles of all ribosomal protein transcripts**. Genes that are transcribed as monocistrons or represent the first gene of the operon are shown as dark blue lines (single genes/1. gene in operon). All other genes are organized in operons and are localized up to 1.2 kb (light blue lines), between 1.3 and 2.4 kb (green lines), between 2.5 and 4.5 kb (orange lines), and ≥4.6 kb (red lines) downstream of the start codon of the first gene of the respective operon. The microarray signal intensity (expression) was normalized to time 0 h. Numbers in parentheses indicate the position within the operon. Genes without numbers in parentheses are monocistronic.

At first glance, cluster 11 also appears to be enriched for genes from three functional groups, the genes of which are organized in large operons: ribosomal protein encoding genes (13 out of 53); ATPase complex encoding genes (5 out of 8); and CO_2 _fixation related genes (5 out of 9). However, detailed investigations revealed an intriguing relationship between half-life and position of these genes within operons, with representatives of cluster 11 being located in the middle to end of their respective operons. Indeed, genes are generally grouped into clusters according to their position within the operon (Additional file 3). Genes showed greater RNA stability the further they were from the transcriptional start site (see Figure 4 for an example of ribosomal proteins). To more stringently investigate the relationship between gene position within the operon and RNA stability, we calculated the distance of the genes to the first start codon of the respective operon and plotted the distance as a function of the half-life (Additional file 4) and the decay rate (Additional file 4), respectively. A highly significant correlation (half-life: Spearman's r = 0.67, *P *≤ 1e^-16^; decay rate: r = 0.64, *P *≤ 1e^-16^) was obtained, supporting the initial finding that RNA stability becomes more pronounced with increasing distance from the promoter. These data indicate that the RNA half-life of a gene is correlated with its position within an operon, although it is unclear whether this phenomenon has impacted gene order in operons. Hence, it can be inferred that protein coding genes involved in the same function or pathway that are organized in operons do not have the same rates of RNA turnover. Similar findings have been reported previously for operon decay in *E. coli *[7], suggesting that the phenomenon may be widespread amongst bacteria. They further suggest that co-regulation of transcription for genes organized in operons is of greater importance than a need for similar decay rates. In the same fashion, these findings may provide an additional explanation for why genes with similar functions are not necessarily arranged in large operons. Two scenarios can be imagined: genes with vastly different half lives - for example, the half-lives for photosystem II genes ranged from 1.1 minutes (*psbH*) to 18.5 minutes (*psbA*); and genes with identical decay profiles - for example, the *recA *and *recN *repair genes (Additional file 2). Both of these types of relative decay rates would not be possible if these genes were organized in operons and the position within an operon dictated relative half-lives of the genes. For pathway genes such as these, we propose that regulation of gene expression by both independent transcription and independent mRNA turnover is more important than the benefit provided by coordinated transcription in operons.

**Figure 5:**
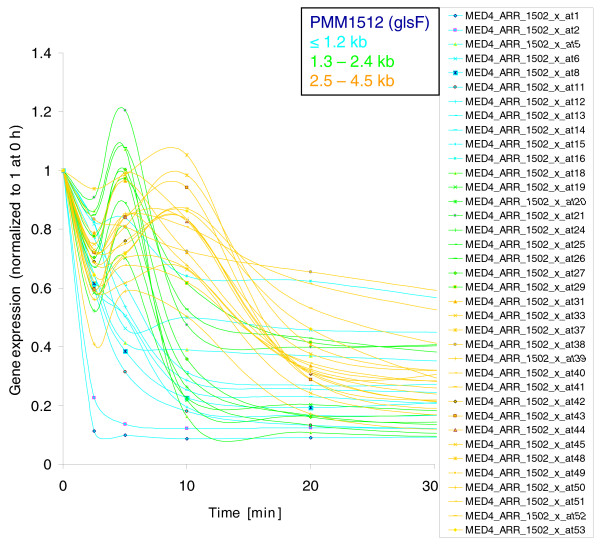
**RNA decay profiles of single probes of *glsF *(ferredoxin-dependent glutamate synthase) - the longest gene (4.6 kb) in *Prochlorococcus *MED4**. Single microarray probes are localized up to 1.2 kb (light blue lines), between 1.3 and 2.4 kb (green lines) and between 2.5 and 4.5 kb (orange lines) downstream of the start codon. The microarray signal intensity (expression) was normalized to time 0 h. Only probes with an expression value above 100 at time 0 h are shown.

The above findings made us wonder whether RNA decay rates are also a function of distance from the transcription start site on a smaller scale, that is, within a gene. We determined half-lives and decay rates of sub-gene segments using single probes for genes at least 2 kb long. Only monocistronic genes and the first gene in an operon were included in this analysis. Even at a single probe level, highly significant relationships were found between the position along the gene and the RNA half-life time and decay rate, respectively (half-life: Spearman's r = 0.65, *P *≤ 1e^-16^; decay rate r = 0.66, *P *≤ 1e^-16^; Figure 5; Additional file 5). These overall findings for large transcripts, whether operons or single genes, further support previous conclusions [7,39,40] that transcript degradation occurs in a 5' to 3' direction.

### Operon decay profiles

**Figure 6:**
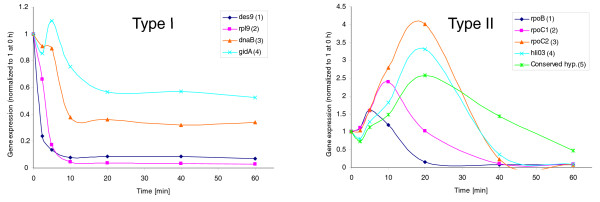
**RNA decay profiles of type I and type II operons**. Both type I (left panel) and type II (right panel) operons have delayed decay profiles that are more pronounced with distance from the promoter. Type I operons are characterized by a plateau in transcript levels prior to decay whereas transcript levels in type II operons increase with time prior to decay and this increase is greater with distance from the promoter. The order of genes within each operon is indicated by numbers in parentheses. The microarray signal intensity (expression) was normalized to time 0 h.

The relationship between the position of a gene in an operon and its half-life suggested complex mRNA decay patterns for operons, leading to an in-depth analysis of their decay profiles that revealed two novel operon decay patterns. Using a comparative genome analysis, Chen *et al. *[41] predicted 88 operons made up of at least 3 genes in *Prochlorococcus *MED4. We used 50 of these for our analysis after removing 24 with weak expression signals (Additional file 6) and another 14 that our data suggest are not likely to be operons (or are operons consisting of only 2 genes). The latter exclusion was based on transcription profiles that are different for individual genes, which is inconsistent with polycistronic messages. Detailed gene expression analysis of the 220 genes within the remaining operons revealed that all operons display one of two distinctive decay profiles (Figure 6). Forty-one operons displayed what we call 'type I' profiles, characterized by a delayed decay profile with increasing distance from the promoter and a temporal plateau prior to transcript decline (Figure 6, left panel). This is particularly obvious for genes in the latter part of the polycistronic message. Nine operons displayed a 'type II' profile, which also had a delayed decay with distance from the promoter, but transcript levels of the latter part of the polycistronic message increased with time and were more pronounced with distance from the promoter (Figure 6, right panel). Therefore longer half-lives of transcript regions further from the transcriptional start site are caused by both a delayed onset of degradation as well as a slower decay rate once degradation begins. From this latter observation and the fact that 3' regions of operons are weakly expressed in general - that is, transcript levels of genes from the distal part of the operon are lower than those of the proximal part - we speculate that the greater stability of transcripts from this region compensates for their relatively low abundance, ensuring that transcripts are available for translation for longer.

**Figure 7:**
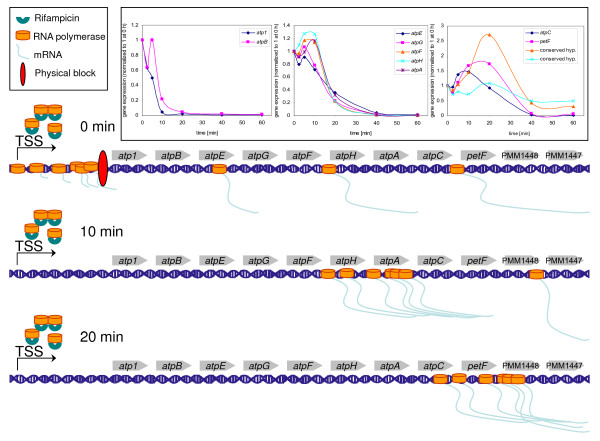
**A possible mechanism of transcriptional delay shown for the type II ATPase operon**. A physical block (red ellipse), which might be built by proteins, congestion of polymerases or convergent polymerases, decelerates the polymerase velocity (0 minutes). After a certain time the block is disintegrated and stalled polymerases can continue with elongation of mRNA (10 minutes and 20 minutes), leading to a relative increase of mRNAs as a function of time and distance. TSS is the transcriptional start site of the operon. The insert on top shows gene expression over time of all genes of the ATPase operon starting with *atp1 *(dark blue line) and ending with PMM1447 (conserved hypothetical in light blue). For better visualization the operon was plotted in three separate graphs. The microarray signal intensity (expression) was normalized to time 0 h.

The atp1BEGFHAC operon, which encodes subunits of the ATPase complex, is a typical example of a type II operon. A temporal increase of up to twofold was found for genes in the more distal section of the operon and in fact the induction level became more pronounced with distance from the promoter (Figure 7). These results were verified by qRT-PCR, which showed an even greater temporal increase in transcript levels for genes furthest from the promoter compared to microarray data (Additional file 2). The rise in transcript level occurred with a considerable delay and may be due to a physical block that is present within the transcription initiation region (Figure 7). Mechanisms for transcriptional interference have been investigated in great detail in *E. coli *(for a review see [42]) and may explain the phenomenon observed here. Shearwin *et al. *[42] provide three plausible explanations for the retardation of the polymerase: model 1, a protein complex of unknown nature sitting downstream of the transcriptional initiation site in the vicinity of the start codon causing a roadblock (Figure 7); model 2, a transcription initiation complex with slower velocity than a polymerase situated upstream and originating from an external promoter (termed 'sitting duck'; we have mapped two transcriptional start sites for the atp1BEGFHAC operon (data not shown) - the primary promoter upstream of *atp1 *and an alternative promoter upstream of *atpE *- which could support this model); and model 3, convergent polymerases may collide, leading to congestion. Sequence data (using 454 technology) of a transcriptome survey show the presence of asRNAs in the operon initiation region (unpublished data), lending support for this latter model at least in this case. Increased half-life times of more distal genes, however, might be the result of the 5' to 3' processivity of endoribonuclease E, the major enzyme during mRNA degradation, and/or *cis*-acting elements coupled with active translation that lead to a stabilization of mRNAs [43]. Secondary structure in the nascent transcripts could also cause such a block. While the aforementioned models may explain type I operon decay profiles, none of them explains the temporal increase in transcript abundance that we observed. It is quite conceivable that more polymerases are sitting in front of the block than polymerase complexes that are still actively involved in elongation. The clearance of the block (caused by its own degradation) could in turn lead to a relative increase of transcript levels due to the release of many polymerase molecules that move as a wave along the operon. The mechanisms described in models 2 and 3 may influence the mRNA stability of the atp operon; however, other mechanisms - for example, model 1 or unknown mechanisms - might also be of importance for the regulation of RNA stability and need to be investigated further to completely explain the modulation of type II operon RNA metabolism.

Thus, we have observed several intriguing genome-wide RNA decay patterns for genes organized in operons. These include: increased stability once decay begins, delayed onset of decay and increased transcript levels after rifampicin addition, as a function of distance from the transcription start site. Although these patterns were not apparent in a similar study of the *Sulfolobus *archaea [11], they are not restricted to *Prochlorococcus*. As mentioned above, Selinger *et al. *[7] reported increased stability with distance from the transcription start site for many operons. They also found an increase in transcript levels after rifampicin addition for a single operon in *E. coli *- that of the tdc operon. Furthermore, several studies have documented segmental differences in RNA half-lives along the atp operon in *E. coli *with very unstable transcripts for the first two genes (*atp1 *and *atpB*), and longer half-lives for the more distal ones [44-46]. Lastly, Ziemke *et al. *[44] measured translation rates of the ATPase subunits after rifampicin treatment by pulse chase experiments and observed an initial induction in signal intensity, which became more pronounced with increasing distance from the promoter. Despite the differences in methodology between the *E. coli *and the *Prochlorococcus *studies, these combined findings suggest that the correlation between decay patterns and position from the transcription start site may be a general phenomenon for genes organized in operons, at least for the eubacteria.

### Rate of RNA polymerase transcription

The fast RNA turnover we found for *Prochlorococcus *made us wonder whether both RNA transcription and RNA degradation are more rapid in this organism relative to other bacteria. The time taken to achieve peak expression between different probes within a single gene can be used to estimate the transcription rate of RNA polymerase. The average polymerase rate of elongation was estimated to be 7.7 (standard error ± 1.1) and 10.3 (standard error ± 3.0) nucleotides per second based on half-lives and decay rates, respectively, with the median *in vivo *velocity of the polymerase estimated to be 4.8 and 4.5 nucleotides per second for the two methods, respectively. The average rate of transcription in *Prochlorococcus *MED4 is remarkably slower than that reported for *E. coli *of 65 to more than 400 nucleotides per second and an average rate of 91 nucleotides per second [47]. However, elongation rates reported by Dennis *et al. *[47] are derived from ribosomal RNA operons, which show a general greater average rate than that of mRNA transcripts [47]. The slow rate of transcription in *Prochlorococcus *MED4 might be in close correlation with the difference in growth rate of the organisms, differences between the composition of the RNA polymerase complex found in cyanobacteria and other eubacteria [48], or differences in methodology used to estimate these rates. However, slow elongation rates might - together with the fact that a high density microarray was used in this study - explain why type I and II operon profiles could be observed.

Collectively, while *Prochlorococcus *has a more rapid RNA turnover, it has remarkably slower rates of RNA transcription relative to other bacteria.

## Conclusions

The global mRNA half-life of 2.4 minutes reported here for *Prochlorococcus *is the shortest measured for any organism, and is the first reported for a cyanobacterium. *Prochlorococcus *grows photoautotrophically and energy is often found in surplus relative to nutrients such as nitrogen and phosphorus, which are vanishingly scarce in the oligotrophic oceans. A rapid RNA turn-over strategy might be advantageous for the recycling of nucleotides to synthesize novel mRNAs, allowing a very rapid response to changing environmental conditions by adjusting transcript amounts on a short time scale - especially in light of the slow growth rate of this organism. Furthermore, we have detected unusual kinetics of RNA degradation for large transcripts and operons in *Prochlorococcus*, which are likely to exist in other bacteria. The complex patterns of large transcript decay reported here indicate that longer half-lives with distance from the promoter are due to a combination of both a delayed onset of decline and a slower decay rate once degradation begins. This would enable more extensive translation of this portion of an operon and may counter, in part, lower transcript levels that often result from reduced transcription of genes positioned far from the promoter.

## Materials and methods

### Culture and experimental growth conditions

*Prochlorococcus *MED4 was grown at 21°C in Sargasso seawater-based Pro99 medium [49] under 30 μmol quanta m^-2 ^s^-1 ^continuous cool white light with a growth rate of 0.325 day^-1^. Triplicate cultures were divided into seven 30 ml subcultures each and 1.9 ml rifampicin added to a final concentration of 150 μg/ml. Rifampicin was dissolved at a concentration of 2.5 mg/ml in Pro99 medium (the limit of its solubility in aqueous solution) to avoid potential negative impacts of organic solvents on *Prochlorococcus *growth. For sampling time point 0 minutes only 1.9 ml Pro99 medium was added. Cells were harvested after 0, 2.5, 5, 10, 20, 40 and 60 minutes of rifampicin treatment by rapid filtration onto Supor-450 membranes. Filters were immersed in 2 ml RNA resuspension buffer (10 mM sodium acetate pH 5.2, 200 mM sucrose, 5 mM EDTA), snap frozen in liquid nitrogen and subsequently stored at -80°C. The filtration was started 45 s before the respective sampling points to account for the time needed for filtration and storage of filters in liquid nitrogen.

We recently found that DMSO does not negatively affect *Prochlorococcus *growth and carried out a limited comparison of expression profiles for cells treated with rifampicin dissolved in water and DMSO. Expression profiles and half-life measures were similar irrespective of the solution used to dissolve the rifampicin (Additional file 7).

### RNA isolation

Total RNA was extracted from cells on filters using a hot-phenol method described previously [24,50]. Total nucleic acids (12 μg) were treated with 6 U DNase (DNA-free, Ambion, Austin, TX, USA) for 60 minutes at 37°C. RNA was precipitated with 1/10 volume 3 M sodium acetate (pH 5.2), 3 volumes ethanol and resuspended in H_2_O at a concentration of approximately 1 μg/μl RNA.

### Real-time PCR

RNA half-life times of 17 genes were independently validated by quantitative real-time PCR employing the identical RNA samples used in the array hybridizations (Figure 3, Table 1).

RNA (300 ng) were DNAse-treated and reverse-transcribed using QuantiTect reverse transcriptase (Qiagen, Hilden, Germany). Samples were DNAse-treated for 2 minutes at 42°C using 2 μl 7 × gDNA wipeout buffer followed by the reverse transcription in a final volume of 20 μl (containing 1 × Quantiscript RT buffer, Mg^2+^, dNTPs, RT primer mix and RNAse inhibitor). Reactions were incubated at 42°C for 15 minutes. The enzyme was inactivated at 95°C for 3 minutes.

qPCR was performed in an Applied Biosystems 7500 Fast Real-Time PCR system using the ABI Power SYBR Green PCR reagents (Foster City, CA, USA). Each 15 μl reaction contained SYBR^® ^Green 1 Dye, AmpliTaq Gold^® ^DNA Polymerase LD, dNTPs with dUTP/dTTP blend, ROX reference, optimized buffer components and 4.5 μl of the reverse transcription reaction in varying dilutions and different primer concentrations (Additional file 8). The reactions were incubated for 2 minutes at 50°C and then 10 minutes at 95°C followed by 40 cycles of 15 s at 95°C, 30 s at 59°C and 30 s at 72°C. After the last cycle, the PCR products were subjected to heat denaturation over a temperature gradient from 60°C to 95°C at 0.03°C s^-1^. All reactions were performed in triplicates for three biological replicates (that is, nine RT-PCR in total). All samples were tested for the presence of residual DNA during quantitative real-time PCR with an RT-minus control.

The real-time PCR data were analyzed using 7500 Fast Real-Time PCR system sequence detection software version 1.4. Data were plotted asnormalized reporter signal, representing the level of fluorescence detected during the PCR process after subtraction of background noise versus cycle number. A threshold was set manually in the middle of the linear phase of the amplification curve. The Ct value (threshold cycle) is defined as the cycle in which an increase in reporter signal (fluorescence) crosses the threshold. The average of Ct values of the triplicate PCR reactions is labeled dCt. The change in geneX cDNA relative to the endogenous standard (RNase P sRNA, *rnpB*) was determined by 2^- [dCt(geneX)-dCt(rnpB)]^, summarized as 2^-ddCt^.

### cDNA synthesis, labeling and microarray hybridization

Labeling, hybridization, staining and scanning were carried out according to Affymetrix protocols for *E. coli *[51] and [24] using 2.5 μg of total RNA on an Affymetrix high density array MD4-9313 made for *Prochlorococcus *MED4. The custom array covers all gene coding regions with a probe pair (match and mismatch) every 80 bases and every 45 bases in intergenic regions in both sense and antisense orientations. Microarray data have been deposited in NCBI's Gene Expression Omnibus (GEO) under accession number GSE17075 [52].

### Normalization

Most normalization methods of microarray data assume that the expression levels of only a subset of genes differ between single arrays. Since our experiment clearly violates this assumption, we performed a systematic comparison of different schemes to select an optimal one. As quality criterion, the Spearman correlation with quantitative RT-PCR data for the 17 genes was used (Additional file 2). In particular, we compared microarray data derived by either Microarray Array Suite (MAS) or robust multi-array analysis methods (as implemented in the Bioconductor package affy). Additionally, different normalization approaches were performed: scaling to the same medium intensity of all genes; scaling to the same medium intensity of spike controls; scaling to the same medium intensity of RNA genes (assumed to be particularly stable); and no subsequent scaling. Remarkably, the robust multi-array analysis processed microarray data with no subsequent scaling achieved the highest concordance with the quantitative PCR standard (that is, a mean Spearman correlation coefficient of 0.83). This shows that the single microarray measurements were highly consistent, and that subsequent scaling introduced experimental variability rather than reducing it.

### RNA half-life and polymerase transcription rate calculations

For the calculation of the RNA half-time, two methods were applied. The first method, termed 'twofold' decay step, was introduced previously by Selinger *et al. *[7]. The half-life time is calculated based on the fit of an exponential decay between the first time point and the earliest successive time point for which a twofold decrease was detected. In contrast to the initially applied fit of an exponential decay using all time points, the 'twofold' algorithm yielded more robust estimates (data not shown). However, we observed that decay of many transcripts showed two distinct phases: either a fast decay followed by a slow decay, or a delay phase (with constant or even increased expression) followed by a rapid decay. Notably, the latter cases were poorly described by the 'twofold' algorithm. We therefore decided to apply a relative two phase decay model for improved estimation of decay times (minutes):

This model is based on the fit of two successive exponential decays to the time series. Thus, we fitted the first decay exponential to the expression values from t = 0 minutes to t = x minutes and the second exponential decay to the expression values from t = x minutes to t = 60 minutes. To choose the time point x (dividing the time series into the two phases), we repeatedly performed the fitting for all possible time points for x and chose the fit with minimal mean square error of the logged data. In cases where the time point of maximal expression was not t = 0 minutes, we used the last time point with maximal expression as the initial time point for the first exponential decay. Thus, the decay rates were calculated relative to the time point of maximal expression. This allows distinguishing effectively between half-life time and decay rate in the calculations.

The rate of RNA polymerase transcription was assessed for expressed genes with a length of at least 2 kb by first calculating the distance between every probe of a probe set and the first probe of this set. The calculated half-life time of every probe of a set was then subtracted from the first probe of the set. The distance(s) and the difference of the half-life between the probes (t) were used to calculate the rate of transcription (v) as a function of v = s/t. Polymerase transcription rates for all of the single probes were averaged and the mean as well as the median were calculated.

### Clustering

Soft clustering was applied to distinguish different expression profiles as implemented in the Bionconductor Mfuzz package and described previously [53]. In brief, the cluster parameter m was set to 2. The number of clusters was chosen to maximize the functional enrichment of gene clusters.

## Abbreviations

asRNA: antisense RNA; DMSO: dimethyl sulfoxide; ncRNA: non-coding RNA; qRT-PCR: quantitative RT-PCR.

## Authors' contributions

CS and DL conceived and carried out the experiments, analyzed the data and wrote the paper. MF performed the microarray analysis and developed the algorithm for decay rate estimations. TR processed the microarrays. RS coordinated and supervised the processing of microarrays. SWC provided project oversight and wrote the paper. All authors read and approved the final manuscript.

## Supplementary Material

Additional file 1 **Table listing RNA half-lives and decay times for the whole transcriptome of *P. marinus *strain MED4**. Standard errors for half-lives and decay times are presented in columns H and J. For the decay times the lower (column K) and upper (column L) bounds of error intervals are also given. Click here for file

Additional file 2 **Figure comparing microarray and quantitative RT-PCR expression profiles for 17 selected genes**. The top panel compares microarray expression signals (MA; [microarray signal intensity of expression]) and quantitative RT-PCR expression signals (qPCR; [normalized to 100% at maximum]) of biological triplicates. The lower panel shows expression profiles for biological triplicates determined by microarrays (red line) and quantitative RT-PCR (black lines; note for series B two samples at time point 2.5 minutes (in grey) are illustrated). Click here for file

Additional file 3 **Table with estimates of half-lives and decay rates of genes organized in operons and their cluster membership**. Click here for file

Additional file 4 **Figure displaying the relationship between the gene position within an operon and (a) half-life or (b) decay rate**. Click here for file

Additional file 5 **Figure showing the relationship between single probe positions of genes with a size of at least 2 kb (monocistrons and first genes in operons) and (a) half-life or (b) decay rate**. Click here for file

Additional file 6 **Table that compares computationally predicted operons from **[41]** with operon assignment based on this study**. Click here for file

Additional file 7 **Figure comparing transcript profiles of cells treated with rifampicin dissolved in water or DMSO**. RNA expression levels were determined by quantitative real-time PCR and compared to microarray data (MA) for transcripts with the regular exponential decay profiles (representing the majority of the transcriptome) that have very short half-lives: **(a) ***recA *and **(b) **PMM1077 and **(c-e) **transcripts from the type II atp operon. Click here for file

Additional file 8 **Table with information on oligonucleotides used for quantitative RT-PCR**. Click here for file
