# Supplementary figures and images for: Short RNA half-lives in the slow-growing marine cyanobacterium Prochlorococcus
Source: Genome Biol. 2010 May 19;11(5):R54. doi: 10.1186/gb-2010-11-5-r54 (PMC2897979; doi:10.1186/gb-2010-11-5-r54)

## Slide 1
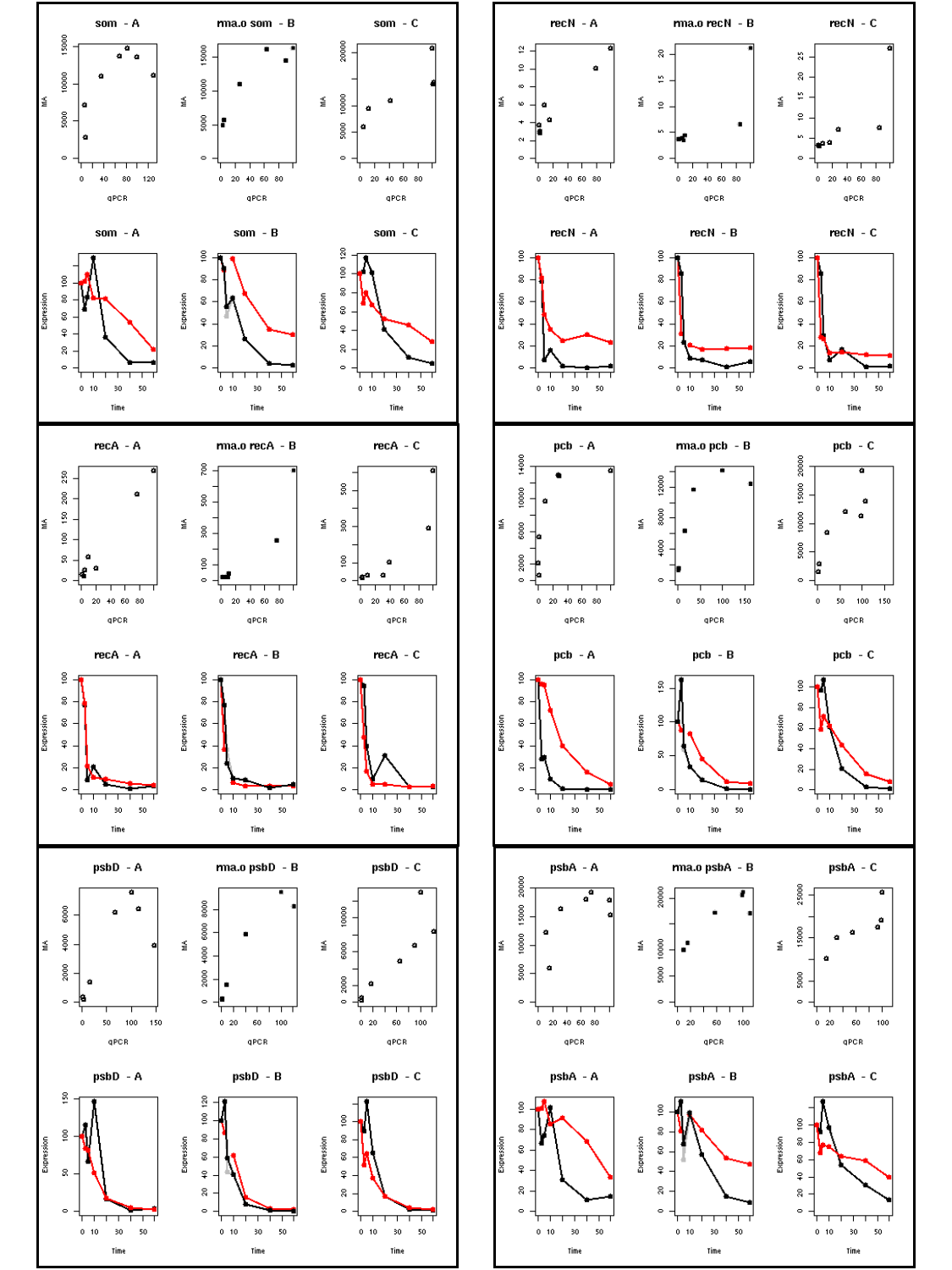

## Slide 2
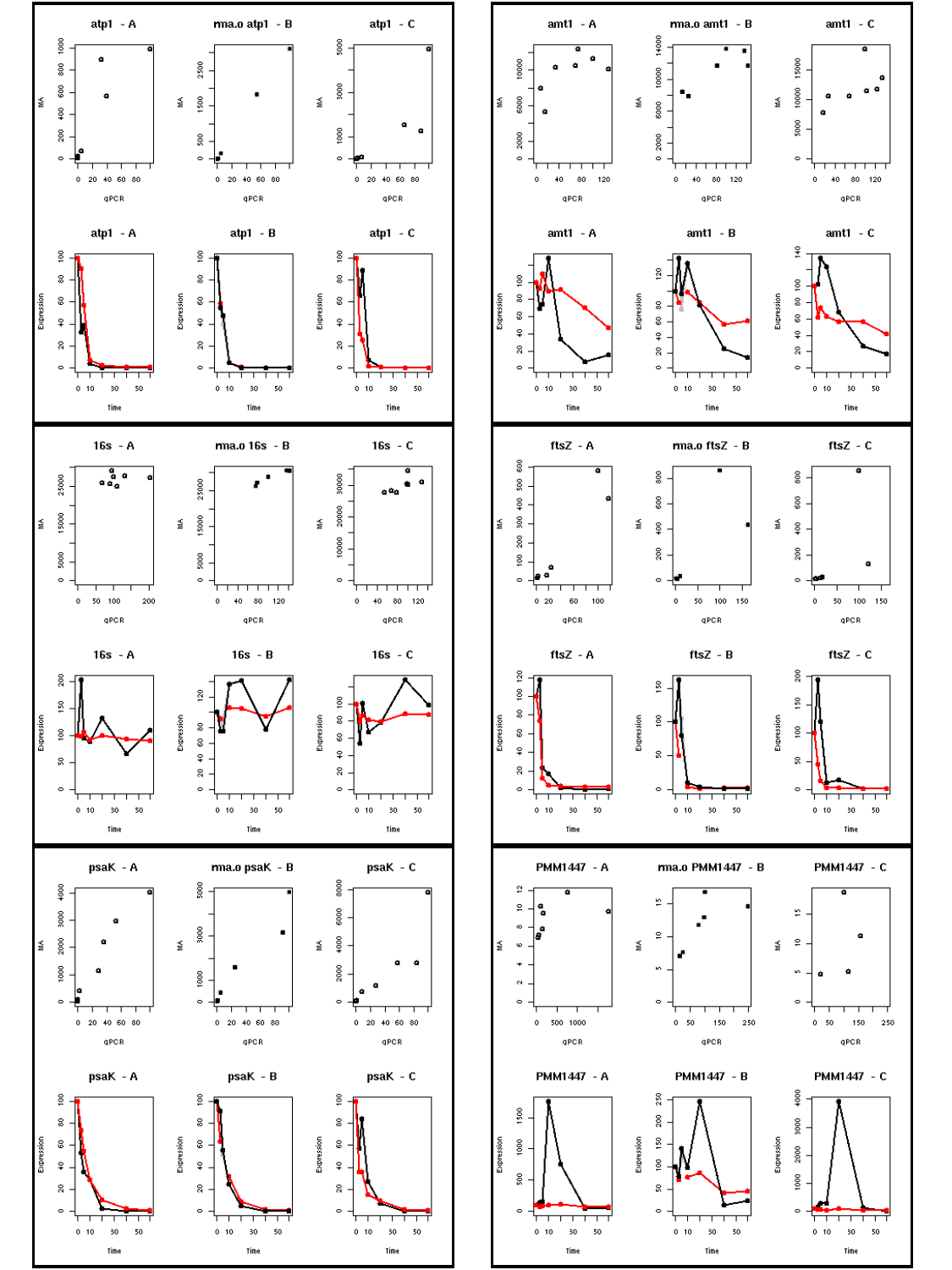

## Slide 3
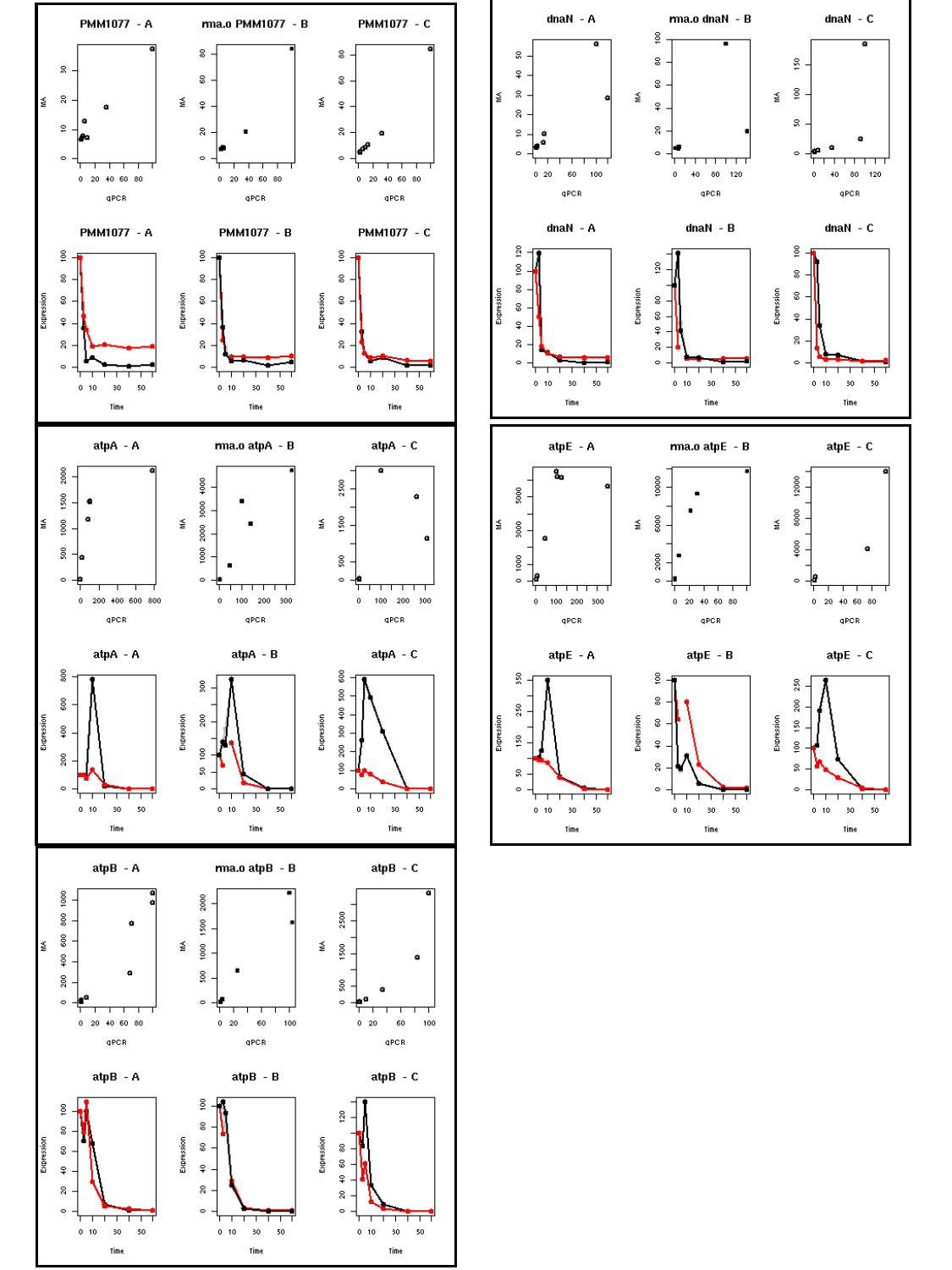

Supplement: Additional file 1 — Table listing RNA half-lives and decay times for the whole transcriptome of P. marinus strain MED4. Standard errors for half-lives and decay times are presented in columns H and J. For the decay times the lower (column K) and upper (column L) bounds of error intervals are also given. [file gb-2010-11-5-r54-S1.PPT]

## Slide 1
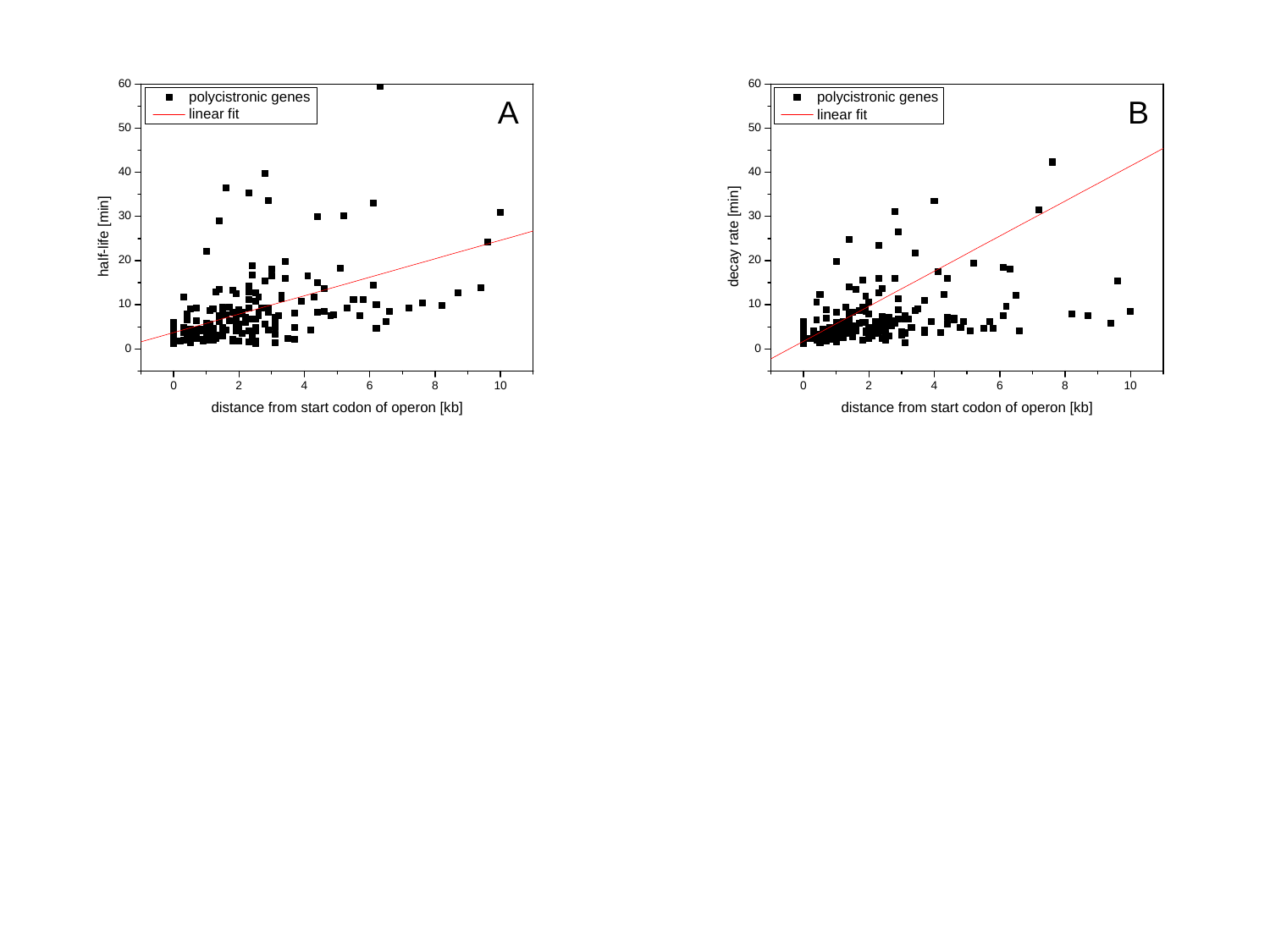

A
B

Supplement: Additional file 2 — Figure comparing microarray and quantitative RT-PCR expression profiles for 17 selected genes. The top panel compares microarray expression signals (MA; [microarray signal intensity of expression]) and quantitative RT-PCR expression signals (qPCR; [normalized to 100% at maximum]) of biological triplicates. The lower panel shows expression profiles for biological triplicates determined by microarrays (red line) and quantitative RT-PCR (black lines; note for series B two samples at time point 2.5 minutes (in grey) are illustrated). [file gb-2010-11-5-r54-S2.PPT]

## Slide 1
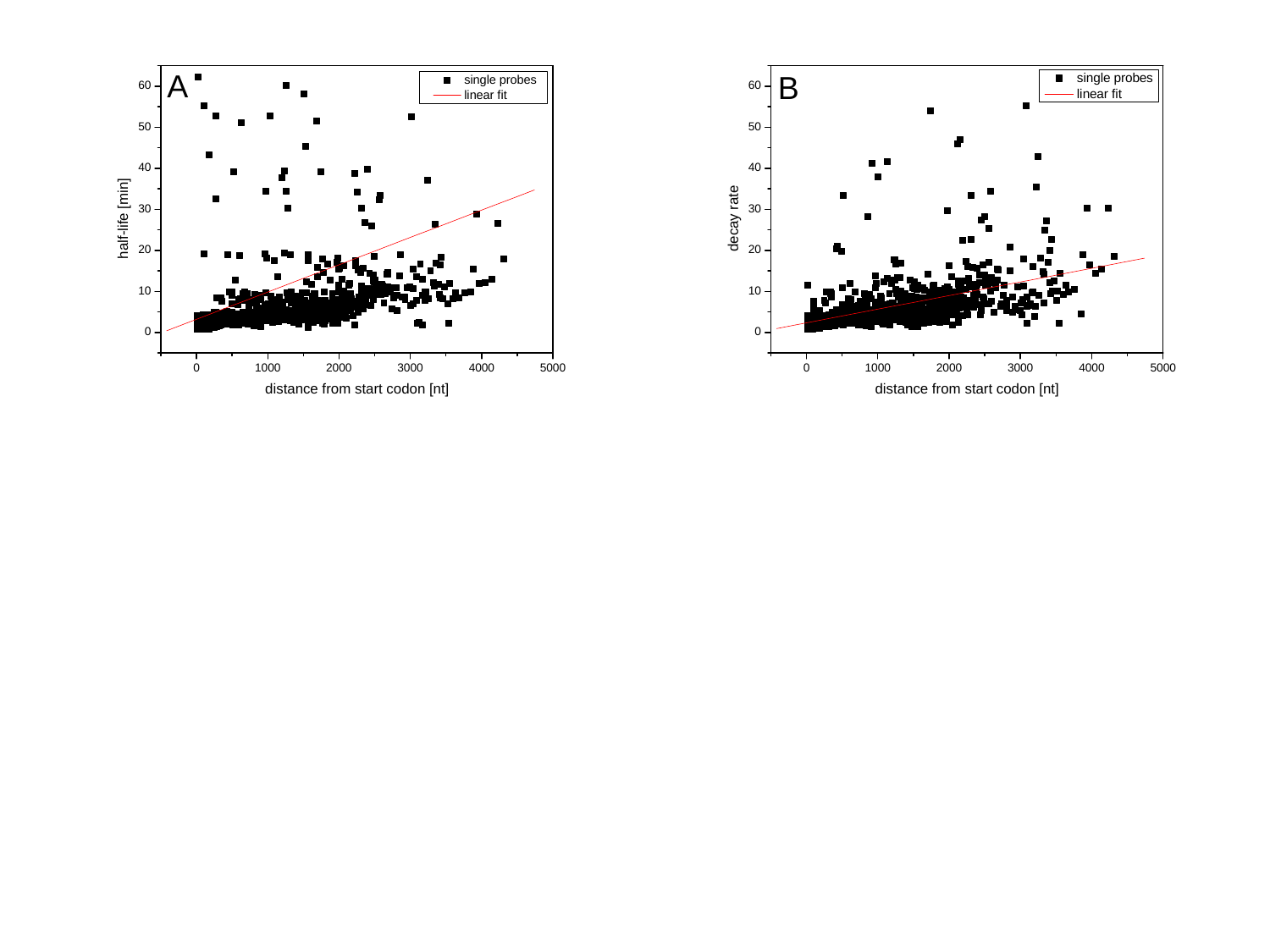

A
B

Supplement: Additional file 3 — Table with estimates of half-lives and decay rates of genes organized in operons and their cluster membership. [file gb-2010-11-5-r54-S3.PPT]

## Slide 1
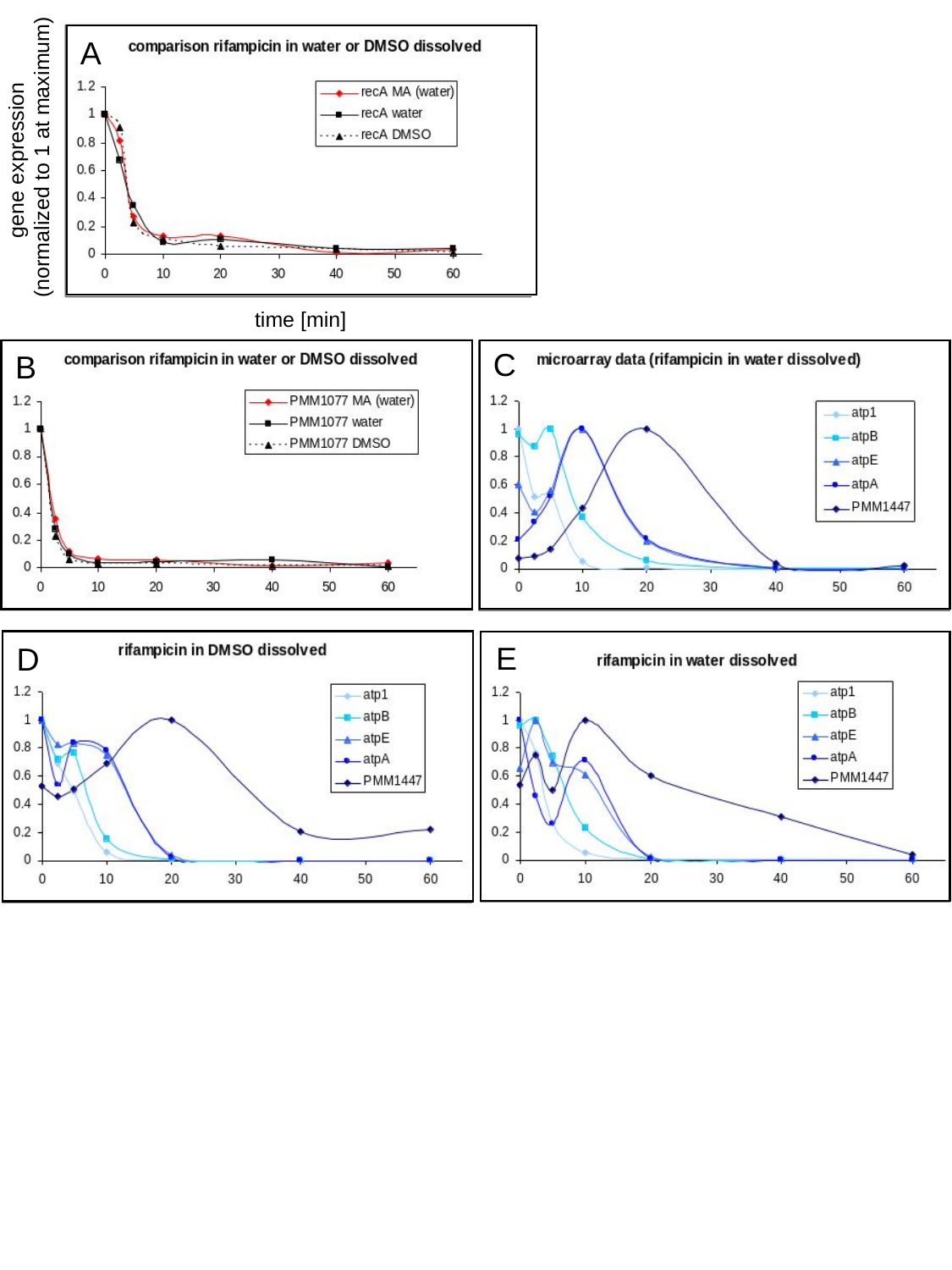

A
gene expression
 (normalized to 1 at maximum)
time [min]
C
B
E
D

Supplement: Additional file 4 — Figure displaying the relationship between the gene position within an operon and (a) half-life or (b) decay rate. [file gb-2010-11-5-r54-S4.PPT]
